# Striatal Pre- and Postsynaptic Profile of Adenosine A_2A_ Receptor Antagonists

**DOI:** 10.1371/journal.pone.0016088

**Published:** 2011-01-11

**Authors:** Marco Orru, Jana Bakešová, Marc Brugarolas, César Quiroz, Vahri Beaumont, Steven R. Goldberg, Carme Lluís, Antoni Cortés, Rafael Franco, Vicent Casadó, Enric I. Canela, Sergi Ferré

**Affiliations:** 1 National Institute on Drug Abuse, IRP, NIH, DHHS, Baltimore, Maryland, United States of America; 2 Centro de Investigación Biomédica en Red sobre Enfermedades Neurodegenerativas (CIBERNED), Faculty of Biology, University of Barcelona, Barcelona, Spain; 3 CHDI Management, CHDI Foundation, Los Angeles, California, United States of America; University of Florida, United States of America

## Abstract

Striatal adenosine A_2A_ receptors (A_2A_Rs) are highly expressed in medium spiny neurons (MSNs) of the indirect efferent pathway, where they heteromerize with dopamine D_2_ receptors (D_2_Rs). A_2A_Rs are also localized presynaptically in cortico-striatal glutamatergic terminals contacting MSNs of the direct efferent pathway, where they heteromerize with adenosine A_1_ receptors (A_1_Rs). It has been hypothesized that postsynaptic A_2A_R antagonists should be useful in Parkinson's disease, while presynaptic A_2A_R antagonists could be beneficial in dyskinetic disorders, such as Huntington's disease, obsessive-compulsive disorders and drug addiction. The aim or this work was to determine whether selective A_2A_R antagonists may be subdivided according to a preferential pre- *versus* postsynaptic mechanism of action. The potency at blocking the motor output and striatal glutamate release induced by cortical electrical stimulation and the potency at inducing locomotor activation were used as *in vivo* measures of pre- and postsynaptic activities, respectively. SCH-442416 and KW-6002 showed a significant preferential pre- and postsynaptic profile, respectively, while the other tested compounds (MSX-2, SCH-420814, ZM-241385 and SCH-58261) showed no clear preference. Radioligand-binding experiments were performed in cells expressing A_2A_R-D_2_R and A_1_R-A_2A_R heteromers to determine possible differences in the affinity of these compounds for different A_2A_R heteromers. Heteromerization played a key role in the presynaptic profile of SCH-442416, since it bound with much less affinity to A_2A_R when co-expressed with D_2_R than with A_1_R. KW-6002 showed the best relative affinity for A_2A_R co-expressed with D_2_R than co-expressed with A_1_R, which can at least partially explain the postsynaptic profile of this compound. Also, the *in vitro* pharmacological profile of MSX-2, SCH-420814, ZM-241385 and SCH-58261 was is in accordance with their mixed pre- and postsynaptic profile. On the basis of their preferential pre- *versus* postsynaptic actions, SCH-442416 and KW-6002 may be used as lead compounds to obtain more effective antidyskinetic and antiparkinsonian compounds, respectively.

## Introduction

The striatum is the major input structure of the basal ganglia [1]. More than ninety five percent of striatal neurons are γ-aminobutyric-acidergic (GABAergic) medium spiny neurons (MSNs). These neurons receive two main inputs: glutamatergic afferents from cortical, thalamic and limbic areas and dopaminergic afferents from the substantia nigra pars compacta and the ventral tegmental area [1]. MSNs are efferent neurons that give rise to the two efferent pathways of the basal ganglia, the ‘direct’ and ‘indirect’ striatal efferent pathways [1]. It is generally accepted that stimulation of the direct and indirect pathways results in motor activation and motor inhibition, respectively, and that smooth motor drive results from the counterbalanced influence of the direct and indirect pathways on the neural activity of the output structures [2], [3]. Direct MSNs express dopamine receptors predominantly of the D_1_ receptor (D_1_R) subtype, whereas indirect MSNs are known for their high expression of dopamine D_2_ receptors (D_2_Rs) and adenosine A_2A_ receptors (A_2A_Rs) [1], [4], [5].

There is clear evidence for the existence of postsynaptic mechanisms in the control of glutamatergic neurotransmission to the indirect MSN by at least two reciprocal antagonistic interactions between A_2A_R and D_2_R [4]. In one type of interaction, A_2A_R and D_2_R are forming heteromers and, by means of an allosteric interaction, A_2A_R counteracts the D_2_R-mediated inhibitory modulation of the effects of NMDA receptor stimulation in the indirect MSN, which includes Ca^2+^ influx, transition to the up-state and neuronal firing in the up-state [6], [7]. This interaction has been suggested to be mostly responsible for the locomotor depressant and activating effects of A_2A_R agonist and antagonists, respectively [4]. The second type of interaction involves A_2A_R and D_2_R that do not form heteromers, but most probably homomers [4]. In this interaction, which takes place at the level of adenylyl-cyclase (AC), stimulation of G_i_-coupled D_2_R counteracts the effects of G_olf_-coupled A_2A_R [4]. Due to a strong tonic effect of endogenous dopamine on striatal D_2_R, this interaction keeps A_2A_R from signaling through AC. However, under conditions of dopamine depletion or with blockade of D_2_R, A_2A_R-mediated AC activation is unleashed. This is biochemically associated with a significant increase in the phosphorylation of PKA-dependent substrates, which increases gene expression and the activity of the indirect MSN, producing locomotor depression (reviewed in ref. [4]). This interaction seems to be the main mechanism responsible for the locomotor depression induced by D_2_R antagonists. Thus the motor depressant and most biochemical effects induced by genetic or pharmacologic blockade of D_2_R are counteracted by the genetic or pharmacological blockade of A_2A_R [8]–[10].

Striatal A_2A_Rs are not only localized postsynaptically but also presynaptically, in glutamatergic terminals, where they heteromerize with A_1_ receptors (A_1_Rs) and where their stimulation facilitates glutamatergic neurotransmission [5], [11]. Interestingly, presynaptic A_2A_Rs are preferentially localized in glutamatergic terminals of cortico-striatal afferents to the direct MSN [5]. According to the widely accepted functional basal circuitry model [2], [3], blockade of postsynaptic A_2A_R localized in the indirect MSN should produce motor activation (by potentiating D_2_R-mediated effects by means of A_2A_R-D_2_R receptor interactions). On the other hand, according to the same model, blockade of presynaptic A_2A_R localized in the cortico-striatal glutamatergic terminals that make synaptic contact with the direct MSN should decrease motor activity (by inhibiting glutamate release). The preferential locomotor-activating effects of systemically administered A_2A_R receptor antagonists can be explained by a stronger influence of a tonic adenosine and A_2A_R receptor-mediated modulation of the indirect pathway *versus* the direct pathway under basal conditions. In any case, the potency at inducing locomotor activation can be used as an *in vivo* measure of the ability of an A_2A_R antagonist to block postsynaptic striatal A_2A_R. Recently we have established an *in vivo* model that evaluates the efficacy of cortico-striatal glutamatergic neurotransmission to the direct MSN, by quantifying the correlation between the current delivered into the orofacial premotor cortex and the concomitant electromyographic response elicited in the jaw muscles [5]. In this model, A_2A_R or D_1_R antagonists were able to counteract the motor output induced by cortical electrical stimulation, which can only be explained by blockade of striatal presynaptic A_2A_R or postsynaptic D_1_R, respectively [5], [12].

Receptor heteromer is defined as a macromolecular complex composed by at least two (functional) receptor units with biochemical properties that are demonstrably different from those of its individual components [13]. Specific ligand binding characteristics are one of those properties [13], [14]. The aim of the present study was, first, to investigate the possible existence of different pre- and postsynaptic profiles of several A_2A_R antagonists. The potency at blocking the motor output and striatal glutamate release induced by cortical electrical stimulation and the potency at inducing locomotor activation were used as *in vivo* measures of pre- and postsynaptic activities, respectively. Second, we wanted to evaluate if the different pre- and postsynaptic profiles could be related to different affinities that A_2A_R could have for those compounds when forming heteromers with either A_1_R or D_2_R. In fact, the results strongly suggest that heteromerization plays a key role in the pre- and postsynaptic profile of A_2A_R antagonists.

## Materials and Methods

### Ethics Statement

All animals used in the study were handled in accordance with the National Institutes of Health Animal care guidelines. The animal research conducted to perform this study was approved by the NIDA IRP Animal Care and Use Committee (under the auspices of protocol 09-BNRB-73) on 12/7/2009.

### Animals

Male Sprague-Dawley rats (Charles River Laboratories, Wilmington, MA) weighting between 300–350 g were used in these experiments. Rats were housed 2 per cage and they maintained at a temperature of 22±2°C on a regular 12-h light–dark cycle. Food and water were available *ad libitum*.

### Adenosine A_2A_R antagonists

The following A_2A_R antagonists were used: 2-(2-Furanyl)-7-[3-(4-methoxyphenyl)propyl]-7H-pyrazolo[4,3-e][1], [2], [4]triazolo[1,5-c]pyrimidin-5-amine (**SCH-442416**), 2-(2-Furanyl)-7-(2-phenylethyl)-7H-pyrazolo[4,3-e][1], [2], [4]triazolo[1,5-c]pyrimidin-5-amine (**SCH-58261**), 2-(2-furanyl)-7-[2-[4-[4-(2-methoxyethoxy)phenyl]-1-piperazinyl]ethyl]-7H-pyrazolo[4,3-e][1], [2], [4]-triazolo[1,5-c]pyrimidin-5-amine (**SCH-420814**), 4-(2-[7-Amino-2-(2-furyl)[1], [2], [4]triazolo[2,3-a][1], [3], [5]triazin-5-ylamino]ethyl)phenol (**ZM-241385**), (E)-1, 3-diethyl-8-(3,4-dimethoxystyryl)-7-methyl-3,7-dihydro-1H-purine-2,6-dione (**KW-6002**), (E)-3-(3-hydroxypropyl)-8-[2-(3-methoxyphenyl)vinyl]-7-methyl-1-prop-2-ynyl-3,7-dihydropurine-2,6-dione (**MSX-2**) and its water-soluble phosphate prodrug (E)-phosphoric acid mono-(3-{8-[2-(3-methoxyphenyl)vinyl]-7-methyl-2,6-dioxo-1-prop-2-ynyl-1,2,6,7-tetrahydropurin-3-yl}propyl) ester disodium salt (MSX-3). **MSX-3** is a water-soluble phosphate pro-drug of MSX-2; in vivo MSX-3 is readily converted to the A_2A_R antagonist MSX-2 (Sauer et al., 2002). For their systemic administration, the compounds were prepared as follows:

SCH-442416 and SCH 58261 were suspended in a solution of 5% dimethyl- sulfoxide (DMSO) (Sigma-Aldrich, St. Louis,MI), 5% TWEEN80 (Sigma-Aldrich, St. Louis, MI) and 90% ddH_2_O; SCH-420814 was suspended in a solution of 20% PEG400, 40% β-cyclodextrin and 40% Lutrol 1% (in ddH_2_O); ZM-241385 was suspended in a solution of 15% DMSO, 10% TWEEN80 and 75% ddH_2_O; KW-6002 was suspended in a solution of 8% TWEEN80 and 92% ddH_2_O; MSX-3 was dissolved in sterile saline (with 3 µl/ml saline of 1 M NaOH solution, final pH 7.4). All drugs but MSX-3 (Sigma-Aldrich, St. Louis, MI) were provided by CHDI Foundation Inc. (Los Angeles, CA, US). SCH-420814 was administered subcutaneously (s.c.) at 1 ml/kg and the other drugs were administered via intraperitonal (i.p.) injection at volume of 2 ml/kg.

### Locomotor Activity

Locomotor activity was measured by placing the animals individually in motility soundproof chambers (50×50 centimeters; Med Associates Inc., VT). Locomotion was measured by counting the number of breaks in the infrared beams of the chambers. The animals were placed in individual acrylic chambers at noon on the day of testing. A lamp inside each chamber remained lit during this period. Following 90 min of habituation, the rats were injected i.p. with different doses of each compound or vehicle and locomotor activity was recorded for 90 min after the drug or vehicle administration. All the animals were tested only once. The effect of different doses of the A_2A_R antagonists on locomotor activity were analyzed using a one-way analysis of variance (ANOVA), followed by Newman-Keuls' *post-hoc* test.

### Surgical procedures

Rats were anesthetized with 3 ml/kg of Equithesin (4.44 g of chloral hydrate, 0.972 g of Na pentobarbital, 2.124 g of MgSO_4_, 44.4 ml of propylene glycol, 12 ml of ethanol and distilled H_2_O up to 100 ml of final solution; NIDA Pharmacy, Baltimore, MD) and implanted unilaterally with bipolar stainless steel electrodes, 0.15 mm in diameter (Plastics One, Roanoke, VA), into the orofacial area of the lateral agranular motor cortex (3 mm anterior, 3 and 4 mm lateral, and 4.2 mm below bregma). The electrodes and a head holder (connected to a swivel during stimulation) were fixed on the skull with stainless steel screws and dental acrylic resin. For the experiments with electromyographic (EMG) recording, electrodes were also implanted in mastication muscles (during the same surgical procedure). Two 5 mm-long incisions were made in the skin on the upper and lower jaw areas to expose the masseter and the lateral pterygoid muscles. Two silicon rubber-coated coiled stainless steel recording electrodes (Plastics One, Roanoke, VA) were slipped below the skin from the incision in the skull until the tips showed up from the incisions in the jaw. The bare tips of the electrodes were then held in contact with the masseter and the lateral pterygoid muscles and the skin was closed with surgical staples. The other end of the recording electrodes was encased in a molded plastic pedestal with a round threaded post which was attached to an electrical swivel and then to a differential amplifier (Grass LP511, Grass Instruments, Warwick, RI). The pedestal was secured to the skull with dental cement together with the stimulation electrodes. For the *in vivo* microdialysis experiments, concentric microdialysis probes with 2-mm long dialysis membranes (Eicom Corp., Tokio, Japan) were implanted respectively into the striatum ipsilateral to the stimulation electrodes (0.0 mm AP, 4.5 ML and 7.0 mm DV).

### EMG recording and power correlation analysis

Rats were placed in individual bowl chambers. Both stimulation electrodes and recording electrodes were attached using flexible shielded cabling to a four channel electrical swivel. Stimulation electrodes were connected to two-coupled constant current isolation units (PSIU6X, Grass Instruments West Warwick, RI) driven by an electrical stimulator (Grass S88X; Grass Instruments). The recording electrodes were connected to a differential amplifier (Grass LP511, West Warwick, RI). This configuration allows the rat to move freely while the stimulation and EMG recordings are taking place. After 60 min of habituation, biphasic current pulse trains (pulse of 0.1 ms at 120–200 µA; 100 Hz, 160 ms trains repeating once per 2 seconds) were delivered. The current intensity was adjusted to the threshold level, defined as the minimal level of current intensity allowing at least 95% of the stimulation pulses to elicit a positive EMG response. Positive EMG response was defined as at least 100% increase of the peak to peak amplitude respect to the background tonic EMG activity lasting more than 100 ms or at least 70% increase in the power of the EMG signal respect to the baseline. Positive EMG responses always matched observable small jaw movements. The threshold level was different for each animal but it was very stable and reproducible once established. The threshold level was in the 100 to 150 µA range for most cases and it reached 200 µA in a few (6) animals. Animals that failed to show a positive EMG response with electrical cortical stimulation intensities of 200 µA were discarded from the experimental procedure (less than 10%). Both stimulator monitoring and the amplified and filtered EMG signal (20,000 times gain, bandwidth from 10 to 1,000 Hz with a notch filter set at 60 Hz) were directed to analog-to-digital converter for recording (Lab-Trax-4, World Precision Instruments, Sarasota, FL) and backup (NI 9215, National Instruments, Austin, TX) and digitized at a sampling rate of 10,000 samples/second. Recordings of the digitized data were made using the software Data Trax2 software (World Precision Instruments) and LabVIEW SignalExpress (National Instruments). A power correlation analysis was used to quantify the correlation between the stimulation pulses of current delivered into the orofacial motor cortex (input signal; µA) and the elicited EMG response in the jaw muscles (output signal; µV). Decrease in the power correlation coefficient (PCC) between these two signals is meant to describe a decrease in the efficacy of the transmission in the neural circuit. Off-line, both signals were rectified and the root mean square (RMS) over each period of the stimulation pulses was calculated in the recorded signals using Data Trax2 software. The transformed data (RMS) from the stimulator monitor and the EMG were then exported with a time resolution of 100 samples/second to a spreadsheet file. The stimulation signal values were used as a reference to select data in a time window of 320 ms starting at the beginning of each train of pulses. This time window was chosen to ensure the analysis of any EMG response whose occurrence or length was delayed from the onset of the stimulation trains and to maximize the exclusion from the analysis of spontaneous jaw movements not associated with the stimulation. Pearson's correlation between the RMS values from the stimulation and EMG signals was then calculated for each experimental subject. PCC was calculated using the data recorded 40 min after the administration of the dose of any compound or vehicle. The effects of the different doses of A_2A_R antagonists on PCC were analyzed by a one-way ANOVA, followed by Dunnett's *post-hoc* test.

### In vivo microdialysis

The experiments were performed on freely moving rats 24 h after probe implantation. An artificial cerebrospinal solution of (in mM) 144 NaCl, 4.8 KCl, 1.7 CaCl_2_, and 1.2 MgCl_2_ was pumped through the microdialysis probe at a constant rate of 1 µl/min. After a washout period of 90 min, dialysate samples were collected at 20-min intervals. After 60 min of collecting samples for baseline, the rats were injected either with the A_2A_R antagonists KW-6002 or SCH-442416. Both compounds were compared to vehicle controls (5% DMSO, 5% of TWEEN80 and 90% of ddH_2_O). After 20 min from drug or vehicle injection, electrical stimulation pulses were applied through the electrodes implanted in the orofacial motor cortex for 20 min (pulse of 0.1 ms at 50–150 µA; 100 Hz, 160 ms trains repeating once × second) and samples were collected for 2 additional hours. Glutamate content was measured by reverse-phase HPLC coupled to a flourimetric detector (Shimadzu Inc., Tokio, Japan) [15]. Glutamate values were transformed as percentage of the mean of the three values before the drug or vehicle injection and transformed values were statistically analyzed. The effect of KW-6002, SCH-442416 and vehicle were analyzed using a one-way ANOVA for repeated measures followed by a Tukey's *post-hoc* test.

### Cell clones

To obtain CHO cells expressing single receptors or co-expressing A_2A_R and A_1_R or A_2A_R and D_2_R, the human cDNAs for A_1_R or D_2_R cloned in pcDNA3.1 vector (containing a geneticin resistance gene) were used. The human A_2A_R was cloned into a pcDNA3.1/Hygro vector with a hygromycin resistance gene. For single transfections, CHO cells were transfected with the cDNA corresponding to A_2A_R, A_1_R or D_2_R using lipofectamine (Invitrogen, Carlsbad, USA) method following the instructions of the supplier. 24 h after transfection the selection antibiotic was added at a concentration that was previously determined by a selection antibiotic test. Antibiotic resistant clones were isolated in the presence of the selection antibiotic (1200 µg/ml geneticin or 1000 µg/ml hygromycin). After an appropriate number of days/passes, several stable lines were selected and cultured in the presence of the selection antibiotic (600 µg/ml geneticin or 300 µg/ml hygromycin). To obtain clones co-expressing A_2A_R and A_1_R or A_2A_R and D_2_R, CHO cells expressing high affinity A_2A_R (obtained as above described) were transfected with the human cDNAs for A_1_R or D_2_R cloned in pcDNA3.1 vector using lipofectamine. After an appropriate number of days/passes stable lines were selected and cultured in the presence of the selection antibiotic. The receptor(s) expression in the cell clones was first detected by dot-blot of cell lysates using commercial available antibodies and wild-type CHO cells lysates as negative basal staining. Positively moderated stained clones were grown to obtain membranes in which the receptor expression was quantified by radioligand-binding experiments (see Results).

### Bioluminescence Resonance Energy Transfer (BRET) assays

The fusion proteins A_2A_R-*Renilla Luciferase* (A_2A_R-RLuc), A_1_R-Yellow Fluorescence Protein (A_1_R-YFP) and D_2_R-YFP were prepared and characterized as described elsewhere [16]. The cDNA encoding serotonin 5HT_2B_-YFP receptor was kindly provided by Dr. Irma Nardi (University of Pisa, Italy). CHO cells were transiently transfected with the corresponding fusion protein cDNA (see Figure legends) using lipofectamine. Cells were incubated (4 h) with the corresponding cDNA together with lipofectamine and Opti-MEM medium (Invitrogen). After 4 hours, the medium was changed to a fresh complete culture medium. Twenty-four hours after transfection, cells were washed twice in quick succession in HBSS with 10 mM glucose and scraped in 0.5 ml of the same buffer. To control the cell number, sample protein concentration was determined using a Bradford assay kit (Bio-Rad, Munich, Germany) using bovine serum albumin dilutions as standards. To quantify fluorescence proteins, cells (20 µg protein) were distributed in 96-well microplates (black plates with a transparent bottom) and fluorescence was read at 400 nm in a Fluo Star Optima Fluorimeter (BMG Labtechnologies, Offenburg, Germany) equipped with a high-energy xenon flash lamp, using a 10 nm bandwidth excitation filter. Receptor-fluorescence expression was determined as fluorescence of the sample minus the fluorescence of cells expressing protein-Rluc alone. For BRET measurements, the equivalent of 20 µg of cell protein were distributed in 96-well microplates (Corning 3600, white plates; Sigma) and 5 µM coelenterazine H (Molecular Probes, Eugene, OR) was added. After 1 minute of adding coelenterazine H, the readings were collected using a Mithras LB 940, which allows the integration of the signals detected in the 485 nm-short- (440–500 nm) and the 530 nm-long-(510–590 nm) wavelength filters. To quantify receptor-Rluc expression luminescence readings were performed after 10 minutes of adding 5 µM coelenterazine H. The net BRET is defined as [(long-wavelength emission)/(short-wavelength emission)]-Cf where Cf corresponds to [(long-wavelength emission)/(short-wavelength emission)] for the Rluc construct expressed alone in the same experiment.

### Radioligand binding experiments

Cells were disrupted with a Polytron homogenizer (PTA 20 TS rotor, setting 3; Kinematica, Basel, Switzerland) for two 5 s-periods in 10 volumes of 50 mM Tris-HCl buffer, pH 7.4 containing a proteinase inhibitor cocktail (Sigma, St. Louis, MO, USA). Cell debris was removed by centrifugation at 1,500 *g* for 5 min at 4°C and membranes were obtained by centrifugation at 105,000 *g* (40 min, 4°C). Membranes were resuspended and centrifuged under the same conditions. The pellet was stored at −20°C, washed once more as described above and resuspended in 50 mM Tris-HCl buffer for immediate use. Membrane protein was quantified by the bicinchoninic acid method (Pierce Chemical Co., Rockford, IL, USA) using bovine serum albumin dilutions as standard. For competition experiments, membrane suspensions (0.2 mg of protein/ml) were incubated for 2 h at 25°C in 50 mM Tris-HCl buffer, pH 7.4, containing 10 mM MgCl_2_ and 0.2 U/ml of adenosine deaminase (ADA, EC 3.5.4.4; Roche, Basel, Switzerland) with the indicated free concentration of the A_1_R, A_2A_R, or D_2_R antagonist [^3^H]DPCPX (GE Healthcare, UK), [^3^H]ZM-241385, or [^3^H]YM-09151-2, respectively (NEN Perkin Elmer, Wellesley, MA, USA) or the A_1_Ragonist [^3^H](R)-PIA (Moravek Biochemicals Inc., Brea, CA, USA) and increasing concentrations of DPCPX, ZM-241385, YM-09151-2, the A_2A_R agonist CGS-21680 or the tested A_2A_R antagonist (all provided by CHDI Foundation Inc.). Non-specific binding was determined in the presence of 11 µM of the corresponding non-radiolabelled ligand. Free and membrane-bound ligand were separated by rapid filtration of 500 µl aliquots in a cell harvester (Brandel, Gaithersburg, MD, USA) through Whatman GF/C filters embedded in 0.3% polyethylenimine that were subsequently washed for 5 s with 5 ml of ice-cold 50 mM Tris-HCl buffer. The filters were incubated with 10 ml of Ecoscint H scintillation cocktail (National Diagnostics, Atlanta, GA, USA) overnight at room temperature and radioactivity counts were determined using a Tri-Carb 1600 scintillation counter (PerkinElmer, Boston, MA, USA) with an efficiency of 62% [17]. All displacers were dissolved in DMSO and diluted in the binding medium. The DMSO concentration in the binding incubates was less than 0.5% and, at this concentration, it did not affect agonist or antagonist affinity for their respective receptors.

### Binding data analysis

Radioligand competition curves were analyzed by nonlinear regression using the commercial Grafit curve-fitting software (Erithacus Software, Surrey, UK), by fitting the binding data to the mechanistic two-state dimer receptor model [18], [19]. Since there is now abundant evidence for GPCR oligomerization, including A_1_R, A_2A_R and D_2_R [20]–[23] and the minimal functional unit of GPCRs in biological tissues seems to imply dimerization [23], this model considers a homodimer as the minimal structural unit of the receptor. Here, we also consider the possibility of a homodimer as the minimal structural unit of a receptor forming homomers or forming heteromers with another receptor. To calculate the macroscopic equilibrium dissociation constants the following equation for a competition binding experiment deduced previously [19], [24] was considered: 
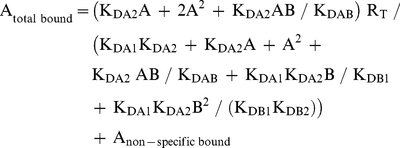
(1)


where A represents free radioligand (the adenosine A_1_R or A_2A_R or dopamine D_2_R antagonist [^3^H]DPCPX, [^3^H]ZM-241385 or [^3^H]YM-09151-2, respectively or the A_1_R agonist [^3^H](R)-PIA) concentration, R_T_ is the total amount of receptor dimers and K_DA1_ and K_DA2_ are the macroscopic equilibrium dissociation constants describing the binding of the first and the second radioligand molecule (A) to the dimeric receptor; B represents the assayed competing compound concentration, and K_DB1_ and K_DB2_ are, respectively, the macroscopic equilibrium dissociation constants for the binding of the first ligand molecule (B) to a dimer and for the binding of the second ligand molecule (B) to the semi-occupied dimer; K_DAB_ is the hybrid equilibrium radioligand/competitor dissociation constant, which is the dissociation constant of B binding to a receptor dimer semi-occupied by A.

When the radioligand A shows non-cooperative behaviour, eq. (1) can be simplified to eq. (2) due to the fact that K_DA2_  =  4K_DA1_ [19], [25] and, therefore, K_DA1_ is enough to characterize the binding of the radioligand A:
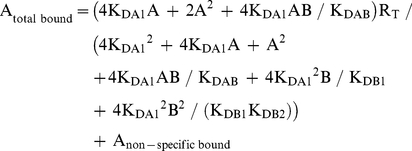
(2)


Binding to GPCRs quite often displays negative cooperativity. Under these circumstances K_D2_/K_D1_>4 and then K_D1_ and K_D2_ represent the “high-affinity” and the “low-affinity” binding sites, respectively. On the other hand, for positive cooperativity, K_D2_/K_D1_<4 and then K_D2_ represents the “high-affinity” and K_D1_ represents the “low-affinity”binding sites [25]. The two-state dimer model also introduces a cooperativity index (D_CB_). The dimer cooperativity index for the competing ligand B is calculated as [19], [25]: 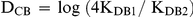



The way the index is defined is such that its value is “0” for non-cooperative binding, positive values of D_C_ indicate positive cooperativity, whereas negative values imply negative cooperativity [14], [19].

In experimental conditions when both the radioligand A and the competitor B (i.e., most adenosine A_2A_ receptor antagonist tested in the present study) show non-cooperativity, it results that K_DA2_  =  4K_DA1_ and K_DB2_  =  4K_DB1_, and eq. (1) can be simplified to: 
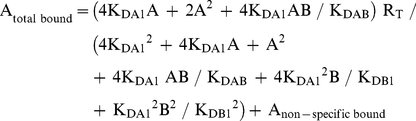
(3)


When both the radioligand A and the competitor B (DPCPX, ZM241385, SCH 23390 or YM-09151-2) are the same compound and the binding is non-cooperative, eq. (3) simplifies to:
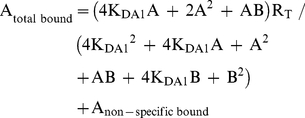
(4)


Goodness of fit was tested according to reduced χ^2^ value given by the nonlinear regression program. The test of significance for two different population variances was based upon the F-distribution (see ref. [25] for details). Using this *F* test, a probability greater than 95% (p<0.05) was considered the criterion to select a more complex equation to fit binding data over the simplest one. In all cases, a probability of less than 70% (p>0.30) resulted when one equation to fit binding data was not significantly better than the other. Results are given as parameter values ± S.E.M. of three-four independent experiments.

## Results

### Striatal pre- *versus* postsynaptic profile of A_2A_ receptor antagonists

**Figure 1:**
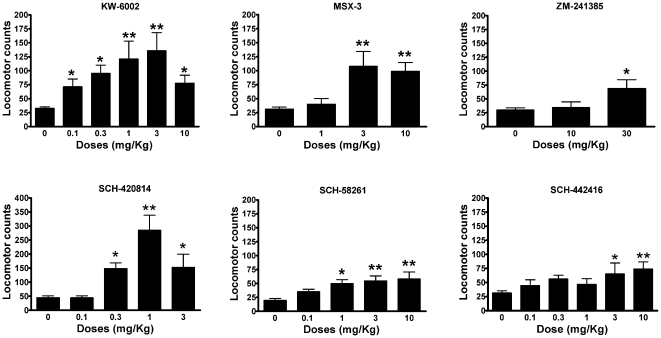
Locomotor activation in rats induced by A_2A_R antagonists. Data represent means ± S.E.M. of the locomotor activity (distance traveled, in cm, of total accumulated counts) in habituated rats (90 min) during 90 min following the drug administration (n = 6–8 per group). * and **: p<0.05 and p<0.01, respectively in comparison to vehicle-treated animals (0 mg/kg); ANOVA with *post-hoc* Newman–Keuls' comparisons, p<0.5 and p<0.01, respectively).

**Figure 2:**
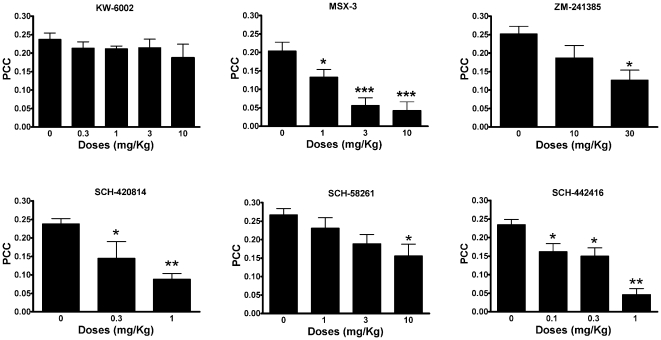
Blockade by A_2A_R antagonists of the motor output induced by cortical electrical stimulation. Dose-dependent decrease in the Power Correlation Coefficient (PCC) induced by the administration of different A_2A_R antagonists. Results represent means ± S.E.M. (n = 5–6 per group). * and **: p<0.05 and p<0.01, respectively in comparison to vehicle-treated animals (0 mg/kg); ANOVA with *post-hoc* Dunnett' comparisons, p<0.5 and p<0.01, respectively).

Dose-response experiments with the six A_2A_R antagonists indicated that four compounds (SCH-420814, SCH-58261, MSX-3 and ZM-241385) had a similar potency (similar minimal significant effective doses) at inducing locomotor activation (Fig. 1) and at reducing PCC (Fig. 2). The other two compounds had a very different profile: KW-6002 produced a strong locomotor activation already at the dose of 0.3 mg/kg i.p., while it did not reduce PCC at the highest tested dose (10 mg/kg i.p.). On the other hand, SCH-442416 produced a very weak locomotor activation, only significant at doses higher than 3 mg/kg i.p., while it significantly decreased PCC already at the dose of 0.1 mg/kg i.p.

**Figure 3:**
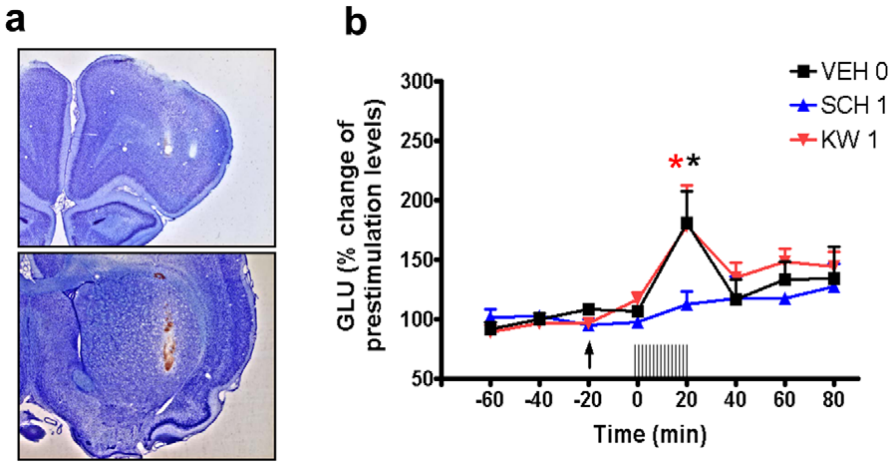
Blockade by A_2A_R antagonists of striatal glutamate release induced by cortical electrical stimulation. (a) Representative coronal sections of a rat brain, stained with cresyl violet, showing the tracks left by the bipolar stimulation electrode in the orofacial area of the lateral agranular motor cortex (top) and by the microdialysis probe in the lateral striatum (bottom). (b) Effect of systemic administration of the A_2A_R antagonists SCH-442416 and KW-6002 (1 mg/kg, i.p., in both cases) on the increase in glutamate extracellular levels in the lateral striatum induced by cortical electrical stimulation. Results are expressed as means ± S.E.M. of percentage of the average of the three values before the stimulation (n = 5–7 per group). Time ‘0’ represents the values of the samples previous to the stimulation. The arrow indicates the time of systemic administration. The train of vertical lines represents the period of cortical stimulation. *: *p*<0.05 compared to value of the last sample before the stimulation (repeated-measures ANOVA followed by Tukey's test).

*In vivo* microdialysis with cortical electrical stimulation was used as an additional *in vivo* evaluation of the preferential pre- and postsynaptic activity of SCH-442416 and KW-6002, respectively. SCH-442416 significantly counteracted striatal glutamate release induced by cortical stimulation at a dose that strongly reduced PCC but did not induce locomotor activation (1 mg/kg i.p.; Fig. 3). On the other hand, KW-6002 did not modify striatal glutamate release induced by cortical stimulation at a dose that produced a pronounced locomotor activation but did not reduce PCC (1 mg/kg i.p.; Fig. 3).

### Development of CHO cell-lines expressing A_1_-A_2A_ or A_2A_-D_2_ receptor heteromers

**Figure 4:**
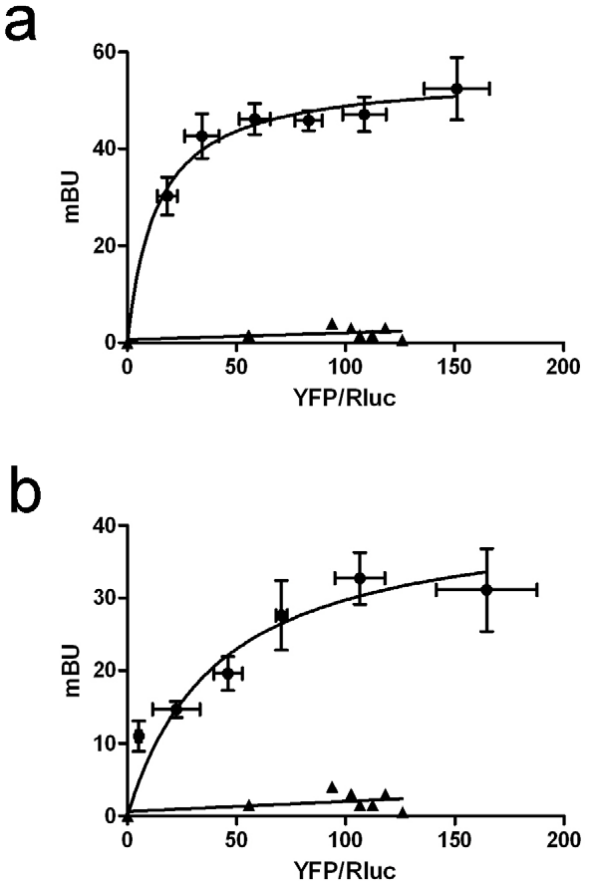
Identification of receptor heteromers in CHO cells by BRET saturation curve. BRET experiments were performed with CHO cells co-expressing A_2A_R-*RLuc* and A_1_R-YFP (A) or A_2A_R-*RLuc* and D_2_R-YFP (B). Co-transfections were performed with increasing amounts of plasmid–YFP (0.25 to 4 µg cDNA corresponding to A_1_R-YFP and 0.5 to 8 µg corresponding to D_2_R-YFP) whereas the A_2A_R-*RLuc* construct was maintained constant (0.5 µg cDNA). Both fluorescence and luminiscence of each sample were measured before every experiment to confirm similar donor expressions (about 100,000 luminescent units) while monitoring the increase acceptor expression (10,000–25,000 fluorescent units). As a negative control, linear BRET was obtained in cells expressing equivalent luminescence and fluorescence amounts corresponding to A_2A_R-*RLuc*, (0.5 µg transfected cDNA) and serotonin 5HT_2B_-YFP (0.5 to 8 µg transfected cDNA) receptors. The relative amount of acceptor is given as the ratio between the fluorescence of the acceptor minus the fluorescence value of cells expressing the donor alone (YFP) and the luciferase activity of the donor (Rluc). BRET data are expressed as means ± S.D. of 4–6 different experiments grouped as a function of the amount of BRET acceptor.

Cell clones expressing A_2A_R, A_1_R-A_2A_R heteromers or A_2A_R-D_2_R heteromers and control clones expressing A_1_R or D_2_R were generated (see Materials and Methods). First of all, the ability of A_2A_R to form heteromers with A_1_R or D_2_R in CHO cells was demonstrated by BRET experiments in cells transiently co-expressing A_2A_R-Rluc and A_1_R-YFP or A_2A_R-Rluc and D_2_R-YFP. A positive BRET signal for energy transfer was obtained (Fig. 4). The BRET signal increased as a hyperbolic function of the concentration of the YFP-fusion construct added reaching an asymptote. As a negative control the BRET pair formed by A_2A_R-Rluc and 5-HT_2B_R-YFP was used. As shown in Figure 4, the negative control gave a linear non-specific BRET signal. The significant and hyperbolic BRET signal found for these fusion proteins indicates that the intermolecular interaction between A_2A_R and A_1_R or A_2A_R and D_2_R in CHO cells is specific.

**Figure 5:**
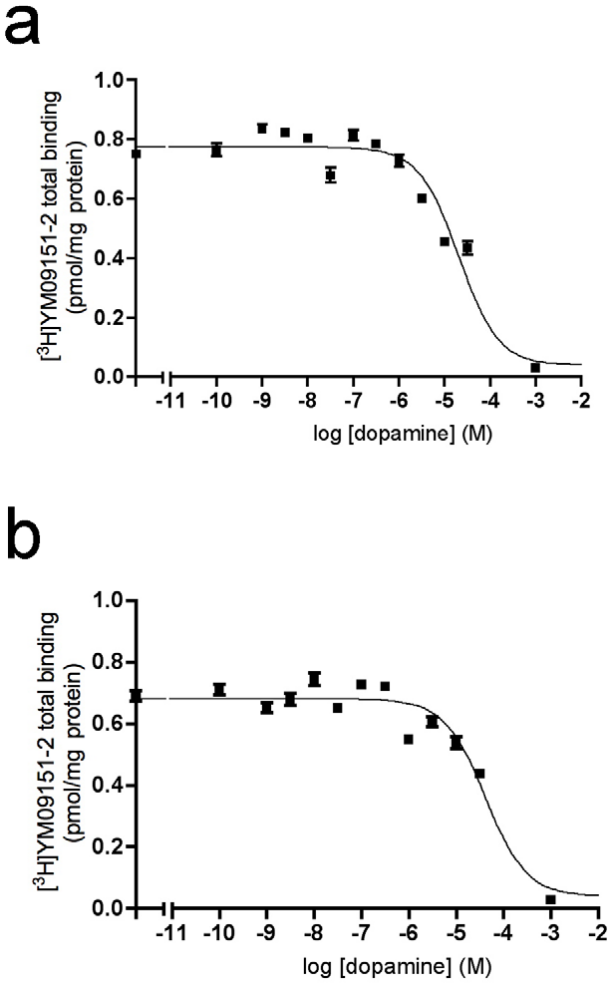
Allosteric interaction between A_2A_R and D_2_R in A_2A_R-D_2_R CHO cells. Competition experiments were performed in membrane preparations from CHO cells expressing A_2A_R and D_2_R with 0.5 nM [^3^H]YM-09151-2 and increasing concentrations of dopamine (from 0.1 nM to 30 µM) in the absence (a) or in the presence (b) of 200 nM CGS-21680 as indicated in Methods. Data represent means ± S.E.M. of a representative experiment performed with triplicates.

**Figure 6:**
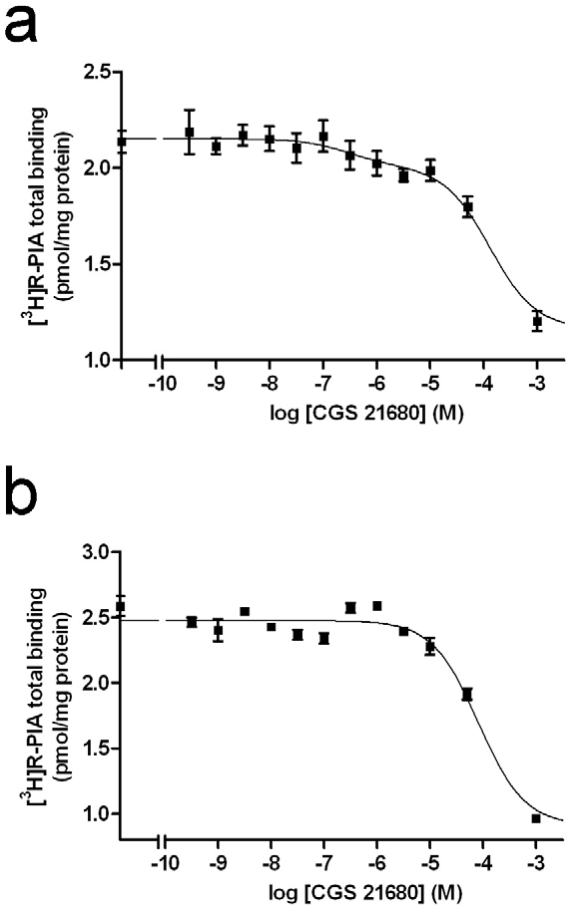
Allosteric interaction between A_1_R and A_2A_R in A_1_R-A_2A_R CHO cells. Competition experiments were performed in membrane preparations from CHO cells expressing A_1_R or A_1_R and A_2A_R with 12 nM [^3^H]R-PIA *versus* increasing concentrations of the A_2A_R agonist CGS-21680 as indicated in Methods. Data represent means ± S.E.M. of a representative experiment performed with triplicates.

A_2A_R-D_2_R and A_1_R-A_2A_R heteromerization in stably transfected CHO cells was shown by ligand binding experiments. This is an indirect approach for the identification of a receptor heteromer in native tissues or cells [13]. In the A_2A_R-D_2_R heteromer, an allosteric interaction between both receptors in the heteromer has been described, in which the dopamine D_2_R agonist affinity decreases in the presence of an A_2A_R agonist [14]. In CHO cells stably expressing A_2A_R and D_2_R, the affinity of the D_2_R for dopamine was determined by competition experiments of the D_2_R antagonist [^3^H]YM-09151-2 *versus* dopamine in the presence (Fig. 5a) or in the absence (Fig. 5b) of the A_2A_R agonist CGS-21680 (200 nM). By fitting data obtained in the absence of CGS-21680 to eq. 3 (Methods; considering K_DA1_ = 2.9 nM see below) the calculated K_DB1_ was 9±2 µM. In the presence of CGS-21680, 5 µM of dopamine was unable to decrease the radioligand bound and more than 50% of radioligand bound was found in the presence of 100 µM of dopamine (Fig. 5b). A K_DB1_ >30 µM was estimated and it was shown that CGS-21680 induced a decrease in the dopamine affinity for D_2_R. An allosteric interaction in the A_1_R-A_2A_R heteromer has also been described, in which the A_1_R agonist affinity decreases in the presence of an A_2A_R agonist [11]. As shown in Figure 6a, the displacement of the A_1_R agonist [^3^H]R-PIA by CGS21680 was significantly (*p*<0.001) better fitted by a biphasic than by a monophasic curve. At low CGS-21680 concentrations, when it binds preferentially to A_2A_R (at concentrations of CGS-21680 <500 nM, the direct binding of CGS-21680 to A_1_R is <1%, according to the calculated affinity of A_1_R for CGS-21680), CGS-21680 decreased the binding of [^3^H]R-PIA to the A_1_R with an IC_50_ value of 386±35 nM (n = 3). At high CGS-21680 concentrations (>10 µM), the [^3^H]R-PIA binding displacement reflects the binding of CGS-21680 directly to the A_1_R and the competition between CGS-21680 and R-PIA for the binding to the A_1_R. In fact, in the control clone expressing only A_1_R, the displacement by CGS-21680 of [^3^H]R-PIA only occurred at CGS-21680 concentrations higher than 10 µM (Fig. 6b).

A pharmacological characterization of selected cell clones was performed with competition experiments of radio-labeled antagonists of A_1_, A_2A_ and D_2_ receptors *versus* selective agonists or antagonists. In all cases, the competition curves of the A_2A_R antagonist [^3^H]ZM-241385 (2 nM) *versus* ZM-241385 (0.1 nM to 11 µM), the D_2_R antagonist [^3^H]YM-09151-2 (0.2 nM) *versus* YM-09151-2 (0.01 nM to 11 µM) or the A_1_R antagonist [^3^H]DPCPX (2 nM) *versus* DPCPX (0.1 nM to 11 µM), were monophasic, indicating the absence of cooperativity (see Materials and Methods). By fitting the binding data to eq. 4 (Materials and Methods), the K_D_ (K_D1_) values obtained for the antagonists ZM-241385 or YM-09151-2 were 8±3 nM and 2.9±0.3 nM, respectively, for the chosen A_2A_R-D_2_R clone, the K_D_ values obtained for the A_1_R and A_2A_R antagonists were 8±2 nM (DPCPX) and 1.8±0.4 nM (ZM-241385), respectively, for the chosen A_1_R-A_2A_R cell clone and the K_D_ value obtained for A_2A_R antagonist (ZM-241385) was 0.9±0.3 nM for the chosen A_2A_R cell clone. Also by fitting the binding data to eq. 4 (Materials and Methods), the K_D_ value obtained for the A_1_R antagonist (DPCPX) was 8.6±0.9 nM for the A_1_R cell clone and the K_D_ value obtained for the D_2_R antagonist (YM-09151-2) was 0.23±0.08 nM for the D_2_R cell clone. These values were then used to determine the affinity constants showed in Tables 1 and 2. The agonists affinity in each selected clone was determined by competition experiments using the A_2A_R antagonist [^3^H]ZM-241385 (2 nM) *versus* the agonist CGS-21680 (1 nM to 50 µM), the D_2_R antagonist [^3^H]YM-09151-2 (0.2 nM), *versus* the agonist quinpirole (0.1 nM to 30 µM), or the A_1_R antagonist [^3^H]DPCPX (2 nM), *versus* the agonist R-PIA (1 nM to 50 µM). As it is shown in Tables 1 and 2, the agonist affinity for A_2A_R in A_2A_R, A_2A_R-D_2_R or in A_2A_R-A_1_R cells is in the same range as that reported for brain striatum or for cells expressing human A_2A_R (between 30 and 250 nM) [7]. Nevertheless, the affinity of the A_2A_R for the selective agonist CGS-21680 was slightly but significantly lower when co-expressed with D_2_R (see Table 2). A_1_R (but not A_2A_R or D_2_R) agonist binding showed negative cooperativity (negative D_CB_ values, see Materials and Methods), both in cells expressing A_1_R and in cells co-expressing A_1_R and A_2A_R (Tables 1 and 2).

**Table 1:** Pharmacological parameters for agonist binding to A_1_R, A_2A_R and D_2_R in A_1_R, A_2A_R and D_2_R CHO cells.

Parameters	A_2A_R cells	A_1_R cells	D_2_R cells
K_DB1_	90±30 nM	13±3 nM	120±60 nM
K_DB2_	360±120 nM	1±0.3 mM	480±240 nM
D_CB_	0	−1.3	0
B_50_	180±60 nM	110±30 nM	240±120 nM

Binding data from competition experiments were fitted assuming that receptors form homodimers, and cooperativity (D_CB_ ≠ 0, fitting to eq. 2; Materials and Methods) or non-cooperativity (D_CB_ = 0, fitting to eq. 3; Materials and Methods) in competitor ligand binding was statistically tested (F test). K_DB1_ and K_DB2_ are, respectively, the equilibrium dissociation constants of the first and second binding of B (the A_1_R, A_2A_R, or D_2_R agonists: R-PIA, CGS-21680 or quinpirole, respectively) to the dimer. D_CB_ is the “dimer cooperativity” index for the binding of the ligand B, and B_50_ is the concentration providing half saturation for B. Data are mean ± S.E.M. values of three experiments.

**Table 2:** Pharmacological parameters for agonist binding to A_1_R-A_2A_R and A_2A_R-D_2_R CHO cells.

Parameters	A_2A_R-D_2_R cells		A_2A_R-A_1_R cells	
	A_2A_R	D_2_R	A_2A_R	A_1_R
K_DB1_	200±40 nM*	1.2±0.6 µM	70±10 nM	0.7±0.3 nM
K_DB2_	0.8±0.4 µM	4.8±2.4 µM	280±40 nM	1.1±0.5 µM
D_CB_	0	0	0	−2.6
B_50_	0.4±0.08 µM	2.4±1.2 µM	140±20 nM	30±10 nM

Binding data from competition experiments were fitted assuming that receptors (also when heteromerizing) form homodimers, and cooperativity (D_CB_ ≠ 0, fitting to eq. 2; Materials and Methods) or non-cooperativity (D_CB_ = 0, fitting to eq. 3; Materials and Methods) in competitor ligand binding was statistically tested (F test). K_DB1_ and K_DB2_ are, respectively, the equilibrium dissociation constants of the first and second binding of B (the A_1_R, A_2A_R, or D_2_R agonists: R-PIA, CGS-21680 or quinpirole, respectively) to the dimer. D_CB_ is the “dimer cooperativity” index for the binding of the ligand B, and B_50_ is the concentration providing half saturation for B. Data are mean ± S.E.M. values of three experiments.

*: p<0.05 compared to K_DB1_ values in A_1_R-A_2A_R and A_2A_R cells (Table 1); one-way ANOVA, followed by Newman-Keuls test.

### Screening of A_2A_R antagonists on cells expressing A_1_-A_2A_ or A_2A_-D_2_ receptor heteromers

**Figure 7:**
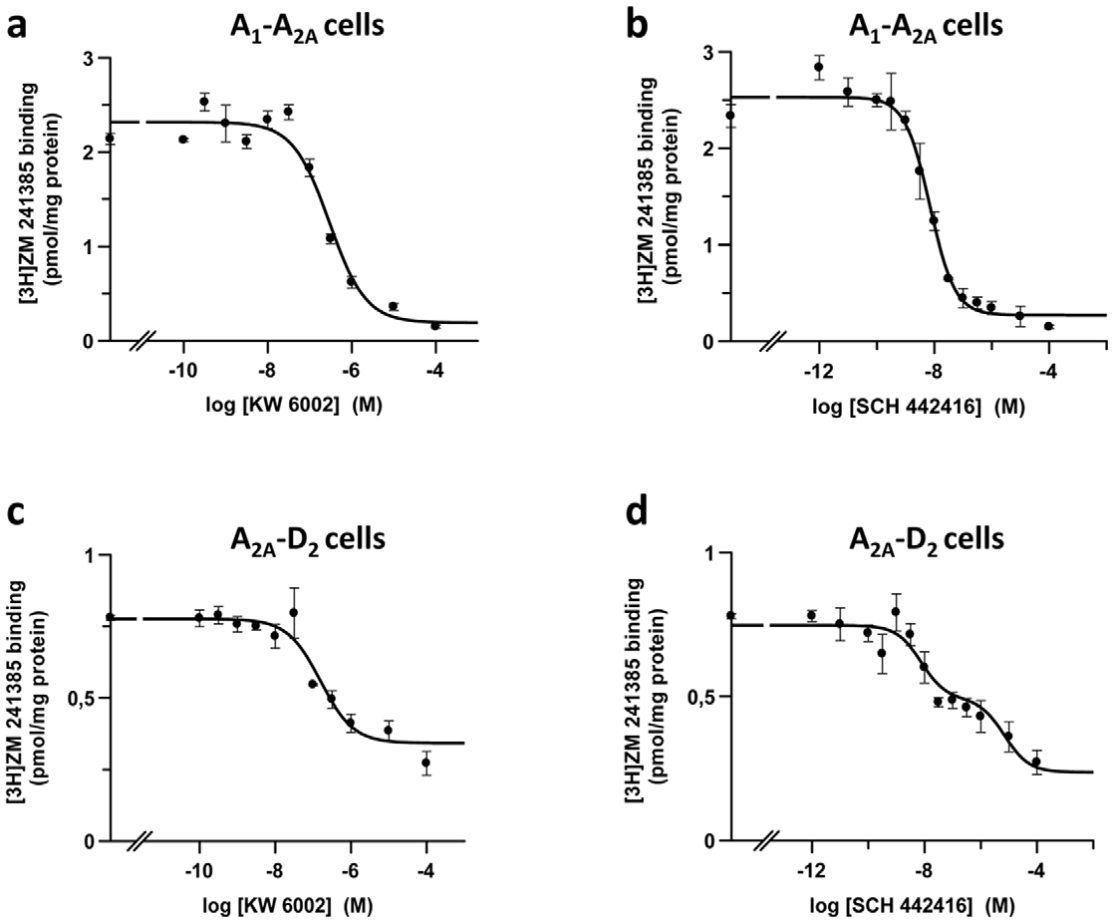
Binding of the A_2A_R antagonists KW-6002 and SCH-442416 to A_1_R-A_2A_R and A_2A_R-D_2_R CHO cells. Competition experiments of [^3^H]ZM-241385 (2 nM) *versus* increasing concentrations of KW-6002 (a and c) or SCH-442416 (b and d) were performed as indicated in Methods in membrane preparations from CHO cells expressing A_1_R and A_2A_R (a and b) or A_2A_R and D_2_R (c and d). Data are means ± S.E.M. of a representative experiment performed with triplicates.

To test if selected A_2A_R antagonists display different selectivity for A_1_R-A_2A_R or A_2A_R-D_2_R heteromers, competition experiments with these ligands were performed using CHO cells expressing A_2A_R, A_1_R-A_2A_R or A_2A_R-D_2_R. We found that none of the six A_2A_R antagonists first tested in the *in vivo* models were able to bind with moderate affinity to A_1_R or to D_2_R in CHO cells expressing A_1_R or D_2_R (data not shown), indicating that these compounds are specific ligands for A_2A_R. Competition experiments of [^3^H]ZM-241385 (2 nM) binding *versus* increasing concentrations of each A_2A_R antagonist (1 nM to 100 µM) were performed as indicated in Methods and binding data from competition experiments were fitted assuming that receptors are dimers and statistically (F test, see Materials and Methods) testing whether the competitor (A_2A_R antagonists) binding was cooperative (biphasic competition curves; fitting to eq. 2) or non-cooperative (monophasic competition curves; fitting to eq. 3). Since the screened compounds are A_2A_R antagonists, competition curves were expected to be monophasic, assuming that antagonist binding is not cooperative. In fact, in all cell clones, MSX-2, KW-6002, SCH-420814, ZM-241385 and SCH-58261 gave monophasic competition curves (fitting binding data to eq. 2 was not better than fitting to eq. 3; see Methods and Fig. 7 a–c as an example). Accordingly, the pharmacological characterization for these compounds gave D_CB_ = 0 and K_DB2_ = 4K_DB1_ (see Table 3). For all compounds, co-transfection with A_1_R did not significantly modify their affinity for A_2A_R. On the other hand, co-transfection with D_2_R significantly reduced the affinity of A_2A_R for MSX-2, SCH-420814, SCH-58261 and ZM-241385, from two to about nine times, and did not significantly modify the affinity of A_2A_R for KW-6002 (Table 3).

**Table 3:** Pharmacological parameters for A_2A_R antagonist binding to A_2A_R, A_1_R-A_2A_R and A_2A_R-D_2_R CHO cells.

K_D1_ (nM)	A_2A_R cells	A_1_R-A_2A_R cells	A_2A_R-D_2_R cells
ZM241385	0.9±0.3	1.8±0.4	8±3*
SCH58261	3.3±0.3	4.7±0.6	23±8*
MSX2	3.2±0.2	4.2±0.3	7±2*
KW6002	100±10	100±20	160±70
SCH420814	0.5±0.1	1.1±0.1	2.7±0.8*

Competition experiments of [^3^H]ZM-241385 (2 nM) binding *versus* increasing concentrations of A_2A_ receptor antagonists were performed as indicated in Methods in membrane preparations from CHO cells expressing A_2A_R or A_1_R and A_2A_R or A_2A_R and D_2_R. Binding data were fitted assuming that receptors (also when heteromerizing) form homodimers, and cooperativity (D_CB_ ≠ 0, fitting to eq. 2; Materials and Methods) or non-cooperativity (D_CB_ = 0, fitting to eq. 3; Materials and Methods) for competitor ligand binding was statistically tested (F test). Only K_DB1_ values (equilibrium dissociation constant of the first binding of B: ZM-241385, MSX-2, SCH-58261, SCH-420814 or KW-6002) are shown, since the analysis demonstrated non-cooperativity for the five A_2A_R antagonists. Data are mean ± S.E.M. values of three experiments.

*: p<0.05 compared to K_DB1_ values in A_2A_R cells; one-way ANOVA, followed by Newman-Keuls test.

For SCH-442416, a careful statistically-based analysis of the monophasic or biphasic nature of the competition curves led to an unexpected finding: in A_2A_R-D_2_R cells, competition curves of [^3^H]ZM-241385 (2 nM) binding *versus* increasing concentrations of SCH-442416 were biphasic (fitting to eq. 2 improves the fitting to eq. 3; see Methods) (Fig. 7d). Table 4 shows the deduced pharmacological parameters from competition experiments of [^3^H]ZM-241385 *versus* SCH-442416 in cells expressing A_2A_R, A_1_R-A_2A_R and A_2A_R-D_2_R. In A_2A_R and A_1_R-A_2A_R cells the curves were monophasic. Accordingly, the pharmacological characterization gave a D_CB_ values of 0 and a K_DB2_ = 4K_DB1_. In contrast, as mentioned above, in cells expressing A_2A_R-D_2_R, competition curves were biphasic, and binding data were then fitted to eq. 2 (Methods) and robust parameters were obtained (Table 4). Thus, in A_2A_R-D_2_R cells, SCH-442416 binding showed a strong negative cooperativity and, consequently, with a marked loss of affinity (an increase of 600 times in K_DB2_) respect to cells expressing A_2A_R. This is reflected by the B_50_ value (concentration competing 50% of radioligand binding), which was more than 40 times higher in A_2A_R-D_2_R cells than in A_1_R-A_2A_R cells or A_2A_R cells.

**Table 4:** Pharmacological parameters for SCH-442416 binding to A_2A_R, A_1_R-A_2A_R and A_2A_R-D_2_R CHO cells.

Parameters	A_2A_R cells	A_1_R-A_2A_R cells	A_2A_R-D_2_R cells
K_DB1_	2.0±0.3 nM	2.4±0.4 nM	7±4 nM
K_DB2_	8±2 nM	10±2 nM	5±2 µM**
D_CB_	0	0	−2.3
B_50_	4.0±0.6 nM	4.8±0.8 nM	190±80 nM**

Competition experiments of [^3^H]ZM-241385 (2 nM) binding *versus* increasing concentrations of SCH-442416 were performed as indicated in Methods in membrane preparations from CHO cells expressing A_2A_R or A_1_R and A_2A_R or A_2A_R and D_2_R. Results were fitted assuming that receptors (also when heteromerizing) form homodimers, and cooperativity (D_CB_ ≠ 0, fitting to eq. 2; Materials and Methods) or non-cooperativity (D_CB_ = 0, fitting to eq. 3; Materials and Methods) of SCH-442416 binding was statistically tested (F test). K_DB1_ and K_DB2_ are, respectively, the equilibrium dissociation constants of the first and second binding of B (SCH-442416) to the dimer. D_CB_ is the “dimer cooperativity” index for the binding of the ligand B, and B_50_ is the concentration providing half saturation for B. Data are mean ± S.E.M. values of three experiments.

**: p<0.01, respectively compared to the K_DB2_ and B_50_ values in A_2_R and A_1_R-A_2A_R cells; Kruskal-Wallis, followed by Dunn's test.

## Discussion

An important finding of the present study is that several A_2A_R antagonists previously thought as being pharmacologically similar present different striatal pre- and postsynaptic profiles. Six compounds already known as selective A_2A_R antagonists were first screened for their ability to block striatal pre- and postsynaptic A_2A_Rs with *in vivo* models. Locomotor activation was used to evaluate postsynaptic activity while PCC reduction was used to determine presynaptic activity (see Introduction). Two compounds, SCH-442416 and KW-6002, showed preferential pre- and postsynaptic profiles, respectively, and four compounds, MSX-3, SCH-420814, SCH-58261 and ZM-241385, showed mixed pre-postsynaptic profiles. Combining *in vivo* microdialysis with cortical electrical stimulation was used as an additional *in vivo* evaluation of presynaptic activity of SCH-442416 and KW-6002. In agreement with its preferential presynaptic profile, SCH-442416 significantly counteracted striatal glutamate release induced by cortical stimulation at a dose (1 mg/kg i.p.) that strongly reduced PCC but did not induce locomotor activation. On the other hand, according to its preferential postsynaptic profile, KW-6002 did not modify striatal glutamate release induced by cortical stimulation at a dose (1 mg/kg i.p.) that produced a pronounced locomotor activation but did not counteract PCC. In a previous study, we reported that intrastriatal perfusion of MSX-3 almost completely counteracted striatal glutamate release induced by cortical electrical stimulation [5], which agrees with its very effective reduction of PCC shown in the present study.

Another important finding of the present study is that at least part of these pharmacological differences between A_2A_R antagonists can be explained by the ability of pre- and postsynaptic A_2A_R to form different receptor heteromers, with A_1_R and D_2_R, respectively [4]–[6], [11], [14]. Radioligand-binding experiments were performed in CHO cells stably expressing A_2A_R, A_2A_R-D_2_R heteromers or A_1_R-A_2A_R heteromers to determine possible differences in the affinity of these compounds for different A_2A_R heteromers. Co-expression with A_1_R did not significantly modify the affinity of A_2A_R for the different ligands, but co-expression with D_2_R decreased the affinity of all compounds, with the exception of KW-6002. The structural changes in the A_2A_R induced by heteromerization with the D_2_R could be detected not only by antagonists but also by agonists. Indeed, the affinity of the selective A_2A_R agonist CGS-21680 was reduced in cells co-transfected with the D_2_R. When trying to explain the differential action of SCH-442416 observed *in vivo*, it is interesting to note that SCH-442416 showed a much higher affinity for the A_2A_R in a presynaptic-like than in a postsynaptic-like context. The binding of SCH-442416 to the A_2A_R-D_2_R heteromer displayed a strong negative cooperativity, phenomenon that was not observed for the binding of SCH-442416 to the A_1_R-A_2A_R heteromer. This negative cooperativity explains the pronounced decrease in affinity of A_2A_R in cells expressing A_2A_R-D_2_R heteromers (B_50_ values 40 times higher in cells expressing A_2A_R-D_2_R than A_1_R-A_2A_R heteromers).

The loss of affinity of A_2A_R upon co-expression of D_2_R was much less pronounced for ZM-241385, SCH-58261, MSX2 or SCH-420814, for which the affinity was reduced from two to about nine fold. Taking into account that these A_2A_R antagonists behave similarly than the A_2A_R agonist CGS-21680 in terms of binding to A_1_R-A_2A_R and A_2A_R-D_2_R heteromers, it is expected that these four compounds compete equally for the binding of the endogenous agonist at pre- and at postsynaptic sites. This would fit with the *in vivo* data, which shows that these compounds have a non-preferred pre-postsynaptic profile. Yet, KW-6002 was the only antagonist whose affinity was not significantly different in cells expressing A_2A_R, A_1_R-A_2A_R heteromers or A_2A_R-D_2_R heteromers. Thus, KW-6002 showed the best relative affinity for A_2A_R-D_2_R heteromers of all coumpounds, which can at least partially explain its preferential postsynaptic profile.

The present results support the notion that receptor heteromers may be used as selective targets for drug development. Main reasons are the very specific neuronal localization of receptor heteromers (even more specific than for receptor subtypes), and a differential ligand affinity of a receptor depending on its partner (or partners) in the receptor heteromer. In the striatum, A_2A_R provides a particularly interesting target, eventually useful for a variety of neuropsychiatric disorders. A_2A_R-D_2_R and A_1_R-A_2A_R heteromers are segregated in different striatal neuronal elements. While A_2A_R-D_2_R heteromers are located postsynaptically in the dendritic spines of the indirect MSNs [4]–[6], [14], A_1_R-A_2A_R receptor heteromers are located presynaptically in glutamatergic terminals contacting the MSNs of the direct pathway [5], [11], [14]. Blocking postsynaptic A_2A_R in the indirect MSN should potentiate D_2_R-mediated motor activation, which is a strategy already used in the development of anti-parkinsonian drugs [26]–[28]. However, blocking A_2A_R in glutamatergic terminals to the direct MSN could potentially be useful in dyskinetic disorders such as Huntington's disease and maybe in obsessive-compulsive disorders and drug addiction [5]. The present results give a mechanistic explanation to the already reported antiparkinsonian activity of KW-6002 [27], [28] and suggest that SCH-442416 could be useful in dyskinetic disorders, obsessive-compulsive disorders and in drug addiction. Medicinal chemistry and computerized modeling should help understanding the molecular properties that determine the particular pharmacological profile of SCH-442416 and KW-6002, which may be used as lead compounds to obtain more effective antidyskinetic and antiparkinsonian compounds, respectively. It will also be of importance to take into account potential changes in the expression of pre- and postsynaptic A_2A_Rs and in their respective heteromers which can occur in those mentioned neuropsychiatric disorders. For instance, dopamine denervation seems to differentially modify the expression of striatal A_2A_R, A_1_R and D_2_R [28]–[31]. This could be addressed by applying the *in vivo* methodology here described to animal models.
